# Wolf cranial morphology tracks population replacement in Fennoscandia

**DOI:** 10.1098/rsos.250358

**Published:** 2025-06-18

**Authors:** Dominika Bujnáková, Jouni Aspi, Carsten Gundlach, Laura Kvist, Christy A. Hipsley

**Affiliations:** ^1^ Ecology and Genetics Research Unit, University of Oulu, Oulu, Finland; ^2^ Department of Physics, NEXMAP, Technical University of Denmark, Lyngby, Denmark; ^3^ Department of Biology, University of Copenhagen, Copenhagen, Denmark

**Keywords:** geometric morphometrics, *Canis lupus*, skull shape, population extirpation

## Abstract

Humans have directly or indirectly contributed to the genetic and thus often phenotypic changes of many species. Anthropogenic pressures, such as persecution and hunting, have negatively affected wolf populations in northern Europe. In line with the genetic replacement that occurred during the twentieth century following the extirpation of wolves from Scandinavia (Norway and Sweden) and their near-extirpation from Finland, we provide evidence of morphological changes in wolf cranial morphology across these populations. Using three-dimensional landmark-based geometric morphometrics, we show that modern wolves in Scandinavia and Finland have, on average, crania with wider frontal bones, wider and higher positioned zygomatic arches and more ventral flexion of the rostrum compared to the historical wolf populations. Although both populations differ in the magnitude and direction of shape change over time, the centroid size or overall size of the cranium, is significantly larger only in the modern Scandinavian wolves. Different genetic origins of the historical and modern populations have probably played a role in the observed morphological variation; however, it is also likely that morphology has been affected by the availability of different prey, which has changed over time.

## 1. Introduction

Morphology is shaped by a complex interplay of genetic, environmental and ecological factors [1–4], with demographic changes further influencing these traits [5–7]. For example, when a population declines to the verge of extinction and begins to recover, genetic and phenotypic characteristics (including morphology) may shift as a result of genetic drift, increased inbreeding, gene flow or replacement owing to immigration [8–10]. Small and fragmented populations are vulnerable to an increased risk of hybridization, which can further alter phenotypic characteristics [1]. Additionally, environmental and ecological factors, such as changes in prey, are directly related to morphology, particularly cranial and dental adaptations [11–14]. Finally, demographic changes exacerbated by climate (e.g. tornadoes, heatwaves) and human activities (e.g. hunting, habitat destruction, species introductions) can further contribute to morphological shifts across populations [3,5–7].

The grey wolf (*Canis lupus*) is a well-known example of a species that has undergone dramatic population declines across the northern hemisphere. Wolves suffered extreme persecution during the nineteenth and twentieth centuries, resulting in their extirpation from half of North America and most of Europe, mainly owing to fearful perceptions of wolves as pests and threats to human life [15]. In Fennoscandia (Norway, Sweden and Finland), negative perceptions of wolves particularly stem from damage caused to reindeer herding, livestock farming and hunting dogs, and were further fuelled by reports of attacks on children dating back to the nineteenth century in Finland. Together, these factors have contributed to continued hunting and poaching pressure [16–19], ultimately causing a bottleneck in the Fennoscandian populations. By the late 1960s, wolves were declared functionally extinct in Scandinavia (hereafter, referring to Norway and Sweden) [20], while Finnish wolves similarly diminished despite migrants from the eastern border with Russian Karelia. In 1915, it was estimated that only 18 (range: 6–54) wolves remained in Finland [21], after which their numbers decreased further during the 1920s and again in the 1970s, marking the time of the lowest population numbers [22–24].

After protection measures were implemented in Fennoscandia (Norway: 1972, Sweden: 1966, Finland: 1973, and increased protection by the European Union in 1995 [19,20]), and a simultaneous increase in their main prey, moose (*Alces alces*; [25,26]), the situation changed. Immigration into the nearly extirpated Finnish population increased, and in the 1980s, the Scandinavian population was re-founded by two immigrant individuals arriving from the east [20,27,28]. Although few migrants arrived in Scandinavia after this date, the offspring of those who did had higher reproductive success than the local inbred wolves originating from the first founders [29]. Despite this immigration, wolf numbers remained low, leading to persistent inbreeding [30,31]. Inbreeding depression soon followed, as evidenced by decreased breeding success [29], increased genetic load [31] and an elevated incidence of congenital pathologies in Scandinavian wolves, which increased in frequency with each year of birth [32]. The Finnish population suffered from low population numbers, largely owing to management hunting and poaching [33], which also contributed to increased inbreeding [34]. This resulted in a genetic replacement of the historical wolves in Scandinavia and a partial replacement in Finland during the twentieth century [24,34–36].

With the onset of the twenty-first century, wolf protection measures, increased forest cover, rural depopulation, and decreased cropland cover, among other factors, have contributed to the recovery of wolves across some of their former ranges in Europe [37,38]. With the reappearance of wolves in Scandinavia after their extirpation, claims emerged that these wolves had been reintroduced by humans, either from a different population or even from zoos [39,40]. A large genomic study did not support this claim [36]. At present, the Scandinavian population numbers 440 individuals (spring 2024) [41] and the Finnish population is estimated at 295 individuals (March 2024) [42]. In addition to genetic changes and an increased incidence of congenital pathologies in modern Scandinavian wolves, the replacement of the Fennoscandian population has probably contributed to changes in morphology. In a study by Engdal [43], some morphological differences between male wolves from historical (extinct, 1830–1972; *n* = 11) and modern (extant, 1983–2018; *n* = 47) Scandinavian populations were observed, based on 16 linear craniometric variables. However, it is still unknown if morphological changes occurred in the Finnish population, how these changes compare between countries, which cranial parts were involved, and how the sexes were affected.

In this study, we investigated whether modern Fennoscandian wolves differ in cranial shape from historical wolves. If so, given genetic evidence for disconnectedness, is the direction and magnitude of morphological change similar for the Finnish and Scandinavian populations? For this, we applied a comprehensive landmarking scheme to three-dimensional models of wolf crania collected from these regions over the past 200 years and analysed the data using geometric morphometric approaches. Additionally, we tested whether samples from zoos differ from wild wolves in cranial shape and if there is an affinity of museum samples without collection dates to the other examined groups.

## 2. Material and methods

### 2.1. Sampling

**Figure 1.**
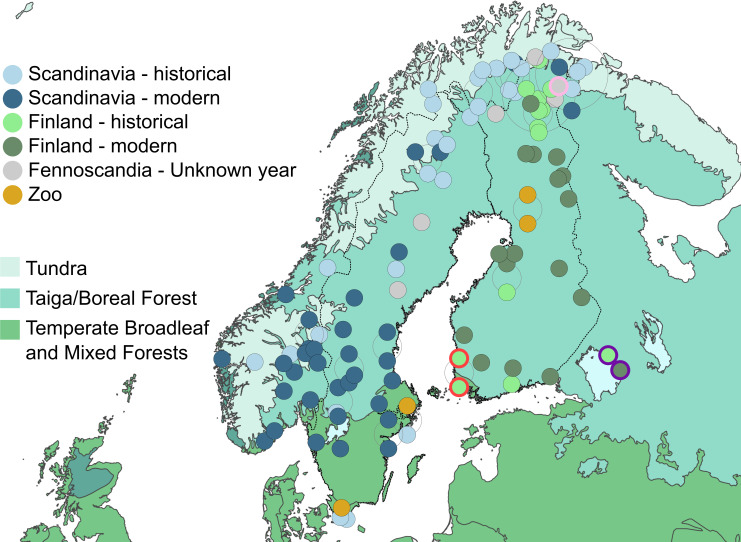
Collection localities of sampled wolf crania across Fennoscandia (Norway, Sweden, Finland and west Russia), with terrestrial ecoregions [45] shown. Samples close to each other are displaced around the locality for better visibility. Sample colours represent groups used for analyses, sample outlines highlight specific individuals of interest: pink and purple outlines represent wolves from northern and southern Russia, respectively, samples with a red outline represent Turku wolves suspected of attacking and killing children. Note that the locations of *zoo* samples indicate the zoos from which they come.

The dataset consists of 84 adult wolf crania from Fennoscandia [44] (Norway, Sweden, Finland and West Russia; see figure 1 with terrestrial ecoregions [45] for sample distribution). Individual ages were not available for all museum records (electronic supplementary material, table S1); therefore, we considered specimens with fully erupted teeth as adults. Seventy-nine specimens were digitized using a Nikon XT H 225 ST X-ray computed tomography (CT) system, of which 72 were scanned at the DANFIX facility of the Danish Technical University (100 kV, 300 uA, 1 s exposure, 1571 projections, two frames per projection, no filter, 120.76 um voxel size), and seven at the Natural History Museum at the University of Oslo (180 kV, 260 µA, 1 s exposure, 3016 projections, one frame per projection, 0.5 mm tin filter, 75.38 µm voxel size). Volumes were reconstructed using X-TEK CT Pro 3D v. XT 4.4.4 Nikon Metrology NV, and three-dimensional surface meshes for each cranium were extracted in VGStudio Max 2.1 (Volume Graphics, Heidelberg, Germany). Three additional specimens were added from Curth *et al*, [46], and two specimens were surface scanned using a Breuckmann SmartSCAN (AICON 3D Systems GmbH, Germany).

To evaluate morphological differences among Fennoscandian wolves in time and space, we split our dataset into geographical (*Finnish* and *Scandinavian*) and temporal (*historical* and *modern*) groups. The geographical division was based on differing demographic and genetic events (§1). These geographical populations are practically isolated from each other because of barriers to dispersal formed by the Baltic Sea in the south and the reindeer herding region in the north (where wolves are routinely killed). The dataset was divided into temporal groups based on dates of lowest population numbers, i.e. the most critical point for each population when genetic turnover took place. The *Scandinavian* population experienced the lowest population numbers in 1966, and the bottleneck continued up to 1983 [20], while the *Finnish* wolves experienced several bottlenecks from the 1920s until the 1970s, with the lowest numbers estimated at the start of the 1920s [24]. For both Scandinavian and Finnish populations, we assumed the first years of the lowest population numbers as the end of the *historical* populations (Scandinavia: 1966, Finland: 1920), with the year after each marking the start of the *modern* populations (Scandinavia: 1967, Finland: 1921).

Claims about the origin of *modern Scandinavian* wolves have suggested that they were released from zoos [39]. Therefore, the dataset also includes four specimens from Fennoscandian zoos, whose origins were traced to mixed ancestry formed by Norwegian, Swedish, west Russian and Estonian wolves [47]. Animals in captivity are known to develop differing morphological characteristics compared to their wild conspecifics [48–50]. Therefore, *zoo* specimens were grouped together. Additionally, five specimens lacked specific collection dates, which is less common in contemporary collections owing to stricter museum policies. We therefore wanted to test if they resemble the *modern* or *historical* wolves, which may be relevant for updating museum records. Hence, they were included as a separate group in the model, *unknown year*. For total sample numbers per group, see table 1.

**Table 1.** Sample information according to year of collection and locality. (Wolf specimens were placed into populations according to the year of the lowest population in the given geographical locality (*Finland* and *Scandinavia*). *Historical* populations end with the year when the lowest population numbers were first recorded, and *modern* populations start the year after. Note that the actual sample years for the *modern* populations start 13 years later for both groups, as the samples from this period were limited while wolf populations experienced bottlenecks.)

population	population temporal boundary	actual sample years	females	males	sex ot available	total *n*
*Scandinavia historical*	until 1966	1800–1959	6	7	11	24
*Scandinavia modern*	from 1967	1980–2018	7	12	5	24
*Finland historical*	until 1920	1854–1914	2	5	5	12
*Finland modern*	from 1921	1934–2009	5	8	1	14
*unknown year*		years not available			6	6
*zoo*		1958, 1959, 2002, 2006	2		2	4
**total**						84

Three west Russian wolf specimens were obtained from European museums: one from the region close to Northern Finland (labelled as a wolf from East Inari, Russia, without a collection year, housed at the Museum für Naturkunde Berlin, Germany) and analysed together with the *unknown year* group; and two from southern parts of the Republic of Karelia, near the Lake Ladoga (1908 and 1943, housed at the Finnish Museum of Natural History, Finland), one analysed with *historical Finnish* specimens and one with *modern Finnish* specimens, respectively. These specimens were relevant for highlighting the source population of the current Fennoscandian wolves.

As part of the *historical Finnish* population, we included two specimens from 1882 that were involved in 22 documented fatal attacks on children in the Turku region, Finland, between 1880 and 1881 [51,52]. Historically, these animals have been described as ‘man-eating wolves’, which referred to the pattern of preying on humans. However, to avoid misinterpretation, we refer to them as ‘Turku wolves’. These specimens are highlighted in graphs, as one of the theories for this behaviour was that these individuals were wolf-dog hybrids [53]. If this is the case, we would expect them to cluster away from the wolf groups.

### 2.2. Morphometric analyses

**Figure 2.**
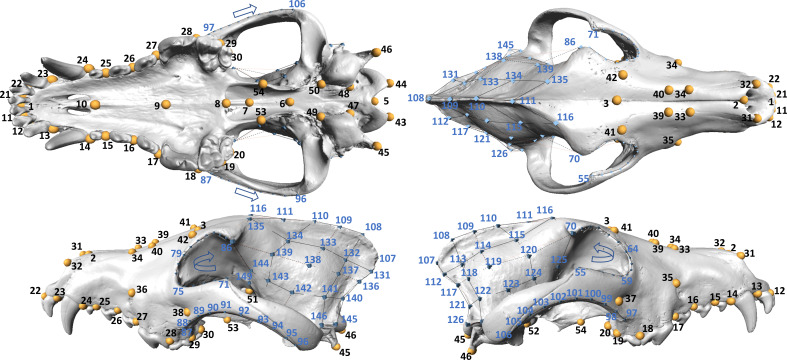
Landmarks used to capture cranial shape. This scheme includes 54-point landmarks (yellow), four curves with 52 sliding landmarks and two surfaces with 43 landmarks (black dots, blue text). In total, 149 landmarks were placed in Stratovan Checkpoint software v.2023 (Stratovan Corporation, Sacramento, USA).

All landmarking was done by one observer (D.B.) in Stratovan Checkpoint v.2023 (Stratovan Corporation, Sacramento, USA), and data were analysed using the *geomorph* package v 4.0.7 [54,55] of R v 4.3.3 [56] (see figure 2 for the placement of landmarks). Out of the 84 specimens, 47 were landmarked twice to assess measurement error. Missing data were estimated using the thin-plate spline method [57], which estimates the locations of missing landmarks in specimens based on a reference specimen obtained from the entire dataset. Although this approach has its drawbacks, as it can pull specimens with large numbers of missing landmarks closer to the mean of the dataset used for estimation, we applied it in order to include incomplete yet important specimens, such as the Turku wolves from Finland.

To obtain shape variables, a generalized Procrustes analysis [58] was performed using the *gpagen* function (further *geomorph* functions will be italicized below), which translated, rotated and scaled individuals to unit centroid size (CS; the square root of the sum of the squared distances of each landmark in the configuration from the centroid). Because crania are symmetric along the midline (and because we do not specifically discuss asymmetry here), the bilateral symmetry component of shape was extracted using the *bilat.symmetry* function for downstream analyses.

We used Procrustes ANOVA to quantify variation in shape and CS among temporal and geographical wolf populations, and between sexes. Statistical significance was obtained through distributions produced by resampling permutations [59,60]. Results were considered statistically significant at *p* < 0.05, and marginally significant for *p*-values between 0.05 and 0.1. A multivariate linear model was used to test for overall shape change over time, and a simple linear model was used to test for shape change along principal component 1 (PC1; representing the greatest variation in the dataset) over time. We also performed analysis on shape residuals after removing the effect of CS, to determine if differences between the groups are a result of differing allometries. We further tested for differences between the allometric slope vector lengths and the correlation between slope vectors and angles. Trajectory analysis was performed to determine the direction of shape change from *historical* to *modern* wolves.

In mammals, sexual dimorphism is widely distributed and has also been reported in wolves, for example in [61–65]; but see [66]. We examined the degree of shape and CS variation explained by sexual dimorphism and whether these differences explain more variation than the division into *modern* and *historical* groups. Because sex was not recorded for all museum specimens, this test was limited to 32 Scandinavian individuals (modern: seven females and 12 males, historical: six females and seven males; table 1).

### 2.3. Visualization

We used principal component analysis (PCA) to visualize shape variation among different groups and the position of individual specimens within morphospace, instead of canonical variates analysis, which is sensitive to overfitting when sample sizes are small. PCA uses aligned shape data obtained after Procrustes superimposition. The resulting shapes are projected onto the eigenvectors, which represent the axes of variance ordered from maximum to minimum as the principal components (PCs). Shape pattern variation along the PC axes was visualized using a three-dimensional warping approach with the *warpRefMesh* and *plotRefToTarget* functions in *geomorph*. Following this approach, an average specimen (determined using *findMeanSpec*) was warped using thin-plate spline to the minima and maxima of the principal components, which were magnified threefold for better visualization of shape differences. Variance in shape between *historical* and *modern Finnish* and *Scandinavian* groups was further visualized using the *rnfelice/hot.dots* function (code available from: https://zenodo.org/records/3929193 [67]). This allowed us to highlight which landmarks, and by extension, which parts of the cranium vary most between groups.

## 3. Results

**Figure 3.**
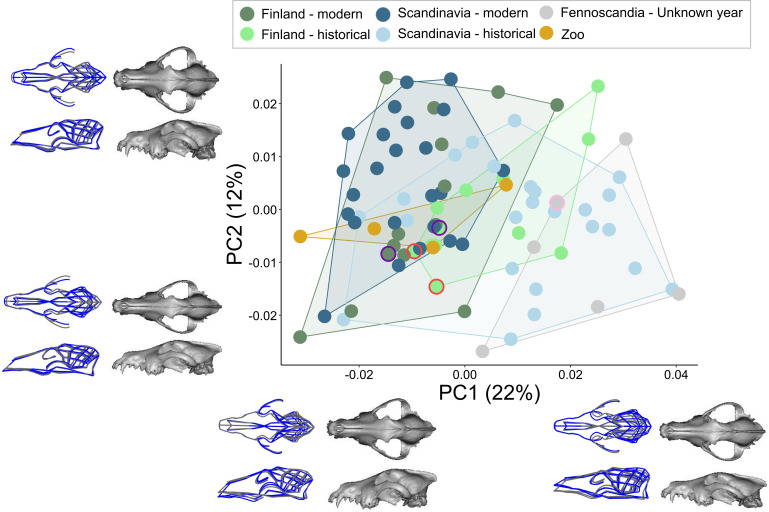
Cranial shape variation along the PC1 and PC2 axes. See the legend for group colours. South Russian wolf specimens are highlighted in purple, north Russian specimens in pink and two Turku wolves are in red. Polygons enclose samples belonging to the same group. Wire frames display shape changes from the mean (grey) to extreme values (blue) at the ends of each PC axis. Both the wire frames and warped cranial images are magnified threefold for better visibility of shape changes.

**Figure 4.**
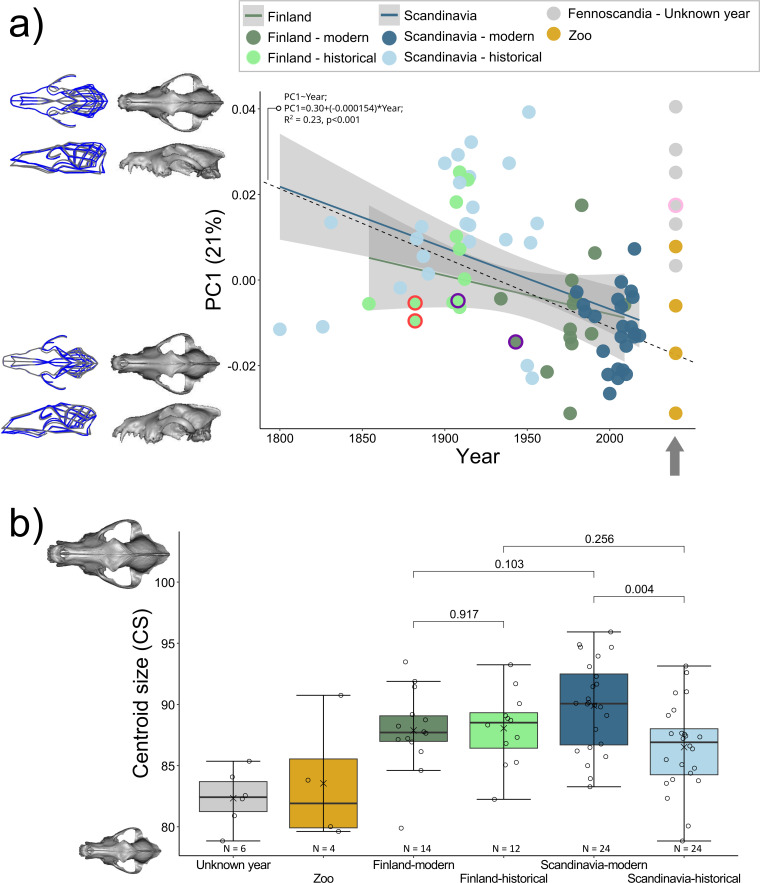
Wolf cranial shape and size variation. (*a*) Temporal shape variation along PC1. Specimens without collection dates and from the zoo are not included in the linear model but are included in the graph and indicated with a grey arrow. Best-fit lines (predicted values) of the linear regression are displayed for Finland and Scandinavia in blue and green, respectively. The black dashed line shows the model with equation for all groups together (excluding zoo samples or samples without year; PCI~year; PC1 = 0.30+(−0.000154)*year; *R*^2^ = 0.23, *p* < 0.001). South Russian wolf specimens are highlighted in dark purple, north Russian specimens in pink and two Turku wolves are in red. (*b*) Change in CS across groups with *p*-values for group comparisons. Note that some groups are represented by few specimens (≤5), hence statistical tests are not presented.

**Figure 5.**
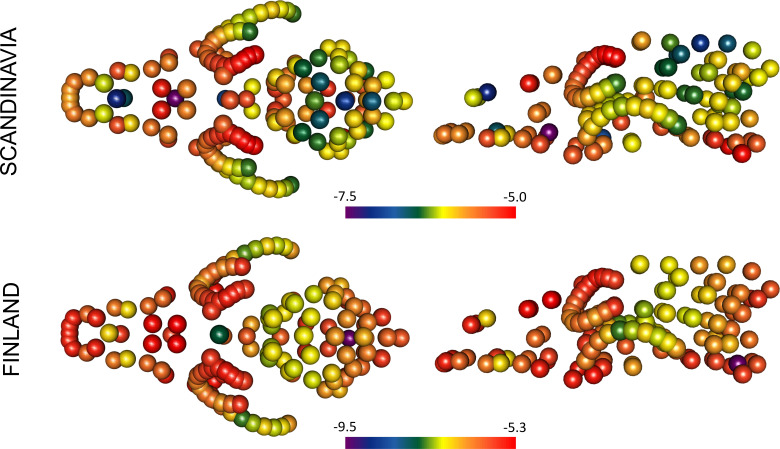
Cranial shape differences between the mean shapes of *historical* and *modern Finnish* and *Scandinavian* wolves, visualized using logarithm-transformed variance. Hotter (red) colours denote higher variance, with landmarks in red highlighting parts of the cranium that vary most between groups. Differences between means largely reflect shape changes in the frontal bones and the ventral part of the cranium. Note that variances were calculated separately for both geographical groups, hence we provide two different scales.

Our results revealed distinct patterns of cranial variation within north European wolves. Measurement error was below 5%, verifying the reliability of our approach (electronic supplementary material, table S2). Upon examination of transformed data, PC1 explained 22% of the variation, representing changes in the width of the frontal bones, the angle of the orbits and part of the rostrum, and changes in the shape of the cranial vault and sagittal crest (figures 3, 4 and 5). On the negative side of PC1, where most of the *modern* specimens group, the rostrum of individuals was sloped further downwards (negative PC1—similar to klinorhynchy vs positive PC1—similar to airorhynchy), frontal bones were wider and the zygomatic arches were positioned higher (more dorsally), the orbits appeared more anterolaterally inclined and the cranial vault was taller. *Historical* specimens mainly fell on the positive side of PC1, showing opposing morphological patterns. PC2, with 12% of the total variation, represents the change in the cranial width, and to a lesser extent than PC1, the change in the width of frontal bones and slope of orbits.

We found evidence for differences in cranial shape and size between the *historical* and *modern* groups, with varying relationships between geographical regions (figures 3–5; table 2; electronic supplementary material, figure S1). *Modern Finnish* (*n* = 14) and *historical Finnish* (*n* = 12) populations differed marginally in mean shape, but not in CS (figures 3 and 4a,b; table 2). *Modern Scandinavian* (*n* = 24) and *historical Scandinavian* (*n* = 24) populations differed in both shape and CS, with high effect sizes (Z) for both (figures 3 and 4a,b; table 2). The crania of *modern Scandinavian* wolves appeared almost 4% larger than *historical* ones. Overall, the morphological differences between the *historical* and *modern* groups were more pronounced in Scandinavia than in Finland. *Historical Finnish* and *historical Scandinavian* groups were no different in shape and CS, while there was a significant difference between *modern Finnish* and *modern Scandinavian* groups (*p* = 0.017, table 2).

**Table 2.** Wolf shape and CS variation across groups. The model includes Sum of Squares (SS), Mean Square (MS), proportion of explained variance (*R*^*2*^), F-statistics (*F*), size effect (*Z*), pairwise Procrustes distance (d), upper confidence interval (UCL), and significance testing (*P*). (Significant and marginally significant differences are shown in bold.)

Fennoscandia *n* = **84**		d.f.	SS	MS	*R* ^2^	*F*	*Z*	*p*
**shape+CS**	CS	1	0.0103	0.0103	0.0972	9.830	5.59	**0.001**
	group	5	0.0150	0.0030	0.1413	2.857	6.39	**0.001**
**shape**	group	5	0.0195	0.0039	0.1834	3.503	6.57	**0.001**
**CS**	group	5	389.59	77.917	0.2913	6.416	3.85	**0.001**
**shape and CS**					**d**	**UCL (95%)**	***Z***	***p***
Finland modern: Finland historical					0.017	0.017	1.64	**0.052**
Finland modern: Scandinavia modern					0.018	0.015	2.54	**0.006**
Finland historical: Scandinavia historical					0.011	0.016	−0.29	0.605
Scandinavia modern: Scandinavia historical					0.025	0.013	4.75	**0.001**
**residual shape variation after accounting for CS**					**d**	**UCL (95%)**	***Z***	***P***
Finland modern: Finland historical					0.018	0.018	1.83	**0.039**
Finland modern: Scandinavia modern					0.017	0.015	2.30	**0.010**
Finland historical: Scandinavia historical					0.011	0.015	−0.40	0.649
Scandinavia modern: Scandinavia historical					0.022	0.013	4.28	**0.001**
**shape only**					**d**	**UCL (95%)**	***Z***	***P***
Finland modern: Finland historical					0.017	0.019	1.23	0.102
Finland modern: Scandinavia modern					0.017	0.016	2.05	**0.021**
Finland historical: Scandinavia historical					0.012	0.017	−0.05	0.511
Scandinavia modern: Scandinavia historical					0.028	0.013	5.16	**0.001**
**CS only**					**d**	**UCL (95%)**	***Z***	***P***
Finland modern: Finland historical					0.175	3.012	−1.388	0.904
Finland modern: Scandinavia modern					2.020	2.632	1.130	0.140
Finland historical: Scandinavia historical					1.541	2.680	0.679	0.284
Scandinavia modern: Scandinavia historical					3.386	2.307	2.264	**0.003**

**Figure 6.**
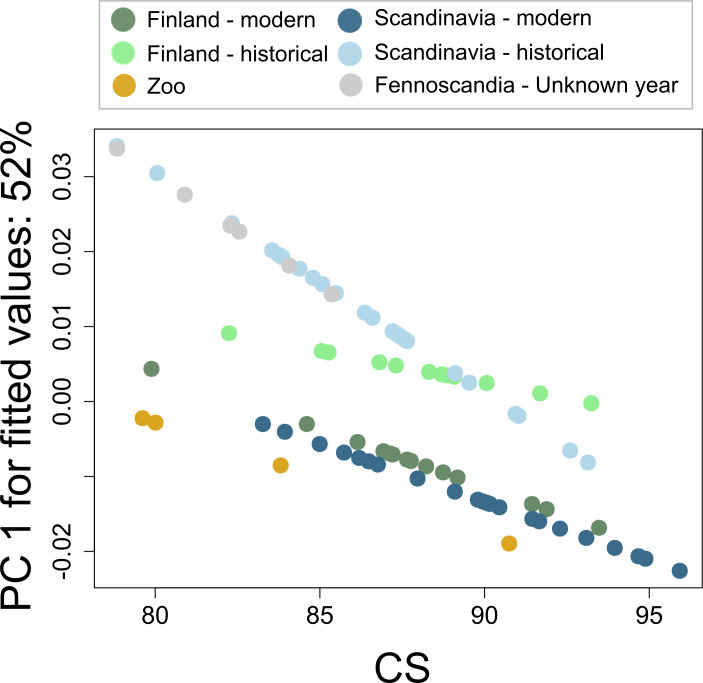
Allometric trends representing a change in shape with increasing CS using the ‘PredLine’ method in the function *plotAllometry* (*geomorph*) using fitted values and CS from the allometric model. Vector lengths for *Scandinavia modern* versus *historical* groups were significantly different (*Z* = 1.64; *p* = 0.041), trend angles were different for *Finnish modern* versus *historical* (*Z* = 2.04; *p* = 0.022), and marginally different for *Scandinavia modern* versus *historical* (*Z* = 1.45; *p* = 0.063). Statistics are not presented for samples lacking year and zoo samples owing to low sample sizes.

**Figure 7.**
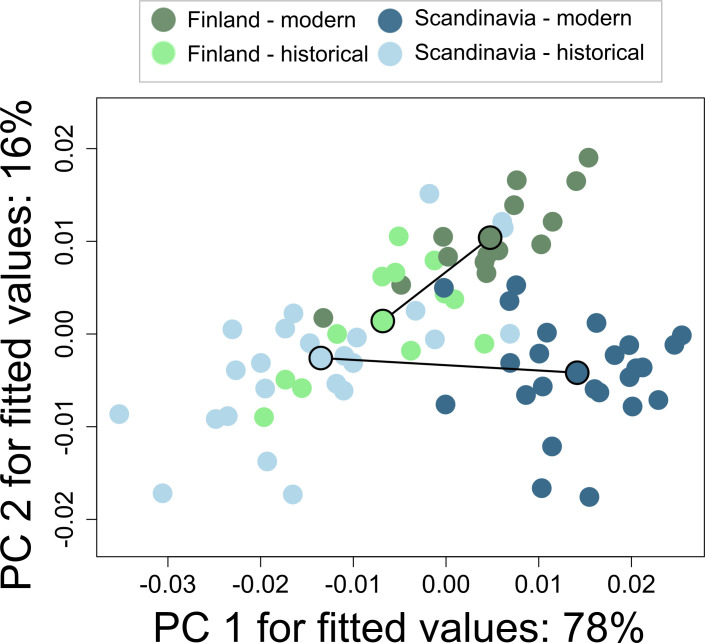
Trajectories of shape change from *historical* (faded colours) to *modern* (darker colours) *Finnish* and *Scandinavian* wolf populations. The path distance for *Scandinavia* is longer (0.0278 versus 0.0177; *Z* = 1.90; *p* = 0.020), and the angle between the trajectories is 54° (*Z* = 3.89; *p* = 0.001).

Although there was a significant effect of CS, after accounting for it in the residual analysis, the differences between the groups remained similar (table 2). Allometric analysis showed a small difference in slope vector lengths between *modern Scandinavian* and *historical Scandinavian* groups (*Z* = 1.64; *p* = 0.041). There was a marginal difference in pairwise angles between slope vectors for *modern Scandinavian* and *historical Scandinavian* groups (*Z* = 1.45; *p* = 0.063), *historical Finnish* and *historical Scandinavian* groups (*Z* = 1.36; *p* = 0.085) and a significant difference between *modern Finnish* and *historical Finnish* groups (*Z* = 2.04; *p* = 0.022; table 3; figure 6). Pairwise analysis of differences in path distances showed a significantly longer path for *Scandinavian* than *Finnish* populations (0.0278 versus 0.0177; *Z* = 1.90; *p* = 0.020), and a significant trajectory angle between *Finland* and *Scandinavia* (angle = 54°; *Z* = 3.89; *p* = 0.001; figure 7).

**Table 3.** Allometry for *modern* and *historical Finnish* and *Scandinavian* populations. The model includes Sum of Squares (SS), Mean Square (MS), proportion of explained variance (*R^2^*), F-statistics (*F*), size effect (*Z*), and significance testing (*P*). (Significant and marginally significant differences are shown in bold.)

Fennoscandia *n* = **74**		d.f.	SS	MS	*R* ^2^	*F*	*Z*	*p*
**allometry**	CS	1	0.008	0.008	0.090	7.966	5.21	**0.001**
**four groups**	group	3	0.010	0.003	0.114	3.346	5.81	**0.001**
	CS*group	3	0.004	0.001	0.047	1.386	1.62	**0.051**
**population pair**		**difference in slope vector lengths**	**effect size**	***p***	**slope vector correlation**	**pairwise angles between slope vectors (in deg.)**	**effect size**	***p***
Finland modern: Finland historical		0.00050	−0.18	0.585	0.05	87	2.04	**0.022**
Finland modern: Scandinavia modern		0.00071	0.31	0.392	0.64	50	−0.36	0.653
Finland historical: Scandinavia historical		0.00006	−1.94	0.971	0.30	73	1.36	**0.085**
Scandinavia modern: Scandinavia historical		0.00112	1.64	**0.041**	0.50	60	1.45	**0.063**

By plotting the PC1 scores against time, a change in shape from historical to modern populations around the 1960s was observed (figure 4a). The clinal relationship of overall shape change with time was significant, where time explained 8% of the total shape variation (*Z* = 4.75, *p* = 0.001; figure 4a). There was a marginal difference between the slopes of the regression models (PC1 versus time) for Finland and Scandinavia (*Z* = 1.58, *p* = 0.069; figure 4a). The samples without a collection year appeared mostly on the positive side of PC1, which included wolves from northern Fennoscandia and northwestern Russia; while samples from the zoo, for which we had the year, but which were not included in the model (since captive animals are known to exhibit differences in morphology), occupied more of the negative side of PC1. A model testing for differences in shape between the groups while accounting for CS showed a significant difference between samples without the collection date (*n* = 6) and *modern Finnish* and *Scandinavian* samples (*p* ≤ 0.008), while this group was not significantly different from the *historical* populations (*p* ≥ 0.374). The samples from the *zoo* (*n* = 4) were significantly different from all the other groups (*p* ≤ 0.07) except from *modern Finnish* wolves (*p* = 0.102).

### 3.1. Sexual dimorphism—Scandinavia

**Figure 8.**
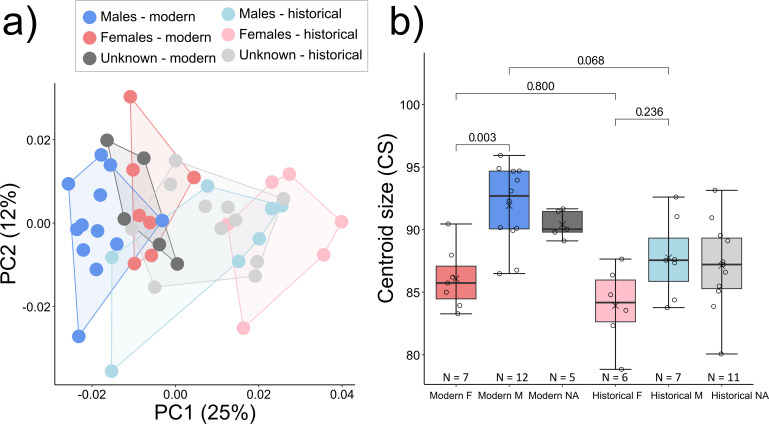
Sexual dimorphism in *historical* and *modern Scandinavian* populations. (*a*) Shape variation along PC1 and PC2, (*b*) CS variation with *p*-values (for models, see table 4).

Sexual dimorphism was observed in the cranial shape of *historical Scandinavian*, but not *modern Scandinavian* wolves (figure 8; table 4). The opposite trend was found for CS, in which sexual size dimorphism was observed only in the *modern Scandinavian* population: *Scandinavian modern* female wolves were on average 6% smaller than male wolves. Note that statistics from Finland for models including sex are not included owing to the small sample sizes of museum specimens with recorded sex (table 1).

**Table 4.** Wolf cranial shape and CS variation across sexes in temporal Scandinavian groups. The model includes Sum of Squares (SS), Mean Square (MS), proportion of explained variance (*R^2^*), F-statistics (*F*), size effect (*Z*), pairwise Procrustes distance (d), upper confidence interval (UCL), and significance testing (*P*). (Significant and marginally significant differences are shown in bold.)

Scandinavia *n* = **48**		d.f.	SS	MS	*R* ^2^	*F*	*Z*	*p*
**shape+CS**	CS	1	0.0081	0.0081	0.1362	8.3695	4.7854	**0.001**
	group	5	0.0117	0.0023	0.1968	2.4205	6.0565	**0.001**
**shape**	group	5	0.0163	0.0033	0.2756	3.1954	5.9662	**0.001**
**CS**	group	5	345.00	69.0000	0.4676	7.3788	3.7557	**<0.001**
**shape and CS (Scandinavia – sex)**					**d**	**UCL (95%)**	***Z***	***p***
females modern: males modern					0.017	0.024	−0.303	0.609
females modern: females historical					0.036	0.024	3.688	**0.001**
males modern: males historical					0.033	0.022	3.430	**0.001**
females historical: males historical					0.026	0.025	1.872	**0.040**
**shape only (Scandinavia – sex)**					**d**	**UCL (95%)**	***Z***	***p***
females modern: males modern					0.018	0.022	0.682	0.256
females modern: females historical					0.037	0.026	3.124	**0.002**
males modern: males historical					0.033	0.023	3.439	**0.001**
females historical: males historical					0.029	0.026	2.161	**0.016**
**CS only (Scandinavia – sex)**					**d**	**UCL (95%)**	***Z***	***p***
females modern: males modern					5.833	3.654	2.550	**0.003**
females modern: females historical					2.157	4.351	0.423	0.800
males modern: males historical					4.156	3.866	1.765	**0.068**
females historical: males historical					3.835	4.449	1.305	0.236

## 4. Discussion

Our results show that cranial shape and size have changed in Fennoscandian wolves over space and time. These observed changes coincided with the genetic replacement of the *historical* wolf populations with the *modern* ones starting in 1966 in Scandinavia [20,28] and during the 1920s up to the 1970s in Finland [23,24]. Undated specimens from museums were most similar in shape to the *historical Fennoscandian* population, thus confirming our expectations based on historical records. Zoo wolves were different from Fennoscandian wolves, supporting previous findings regarding differences between captive and wild individuals [48–50]. Their marginal distinction from the Finnish modern group could be attributed to an eastern origin of those wolves, which also contributed to the Scandinavian zoo population. For a subset of the *Scandinavian* groups with known sexes, we found evidence of sexual dimorphism in the shape of the *historical* wolves, but not in the *modern* population. By contrast, only the *modern* population showed sexual size dimorphism in CS, with *modern* male wolves being significantly larger than *modern* females. Given that sexual dimorphism in wolves is a widespread phenomenon, the likely explanation for this discordance is the small and uneven sample sizes of the *Scandinavian* populations (table 1), which could have resulted in insufficient power to differentiate between males and females.

The extirpation of wolves from Scandinavia during the twentieth century, followed by the limited number of founders and migrants, contributed to the reduced genetic diversity within the *Scandinavian modern* population [30]. By contrast, *Finnish* wolves did not suffer complete local extinction, which together with a larger number of migrants from the east to Finland, contributed to higher genetic diversity compared to Scandinavia [68,69]. A founder effect in *modern Scandinavian* wolves, together with continuous hunting, could explain the difference in the directions and magnitude of shape change between the Finnish and Scandinavian populations. It could also partially account for the significant difference in the lengths of allometric vectors between *historical* and *modern Scandinavian* populations, where the modern population had lower variation in size. However, it is important to note that the variation in allometric vector lengths can also be influenced by differences in the size distribution across groups and in this case may also be influenced by the shorter sampling period for modern populations relative to the historical ones.

While genetic processes such as directional selection, gene flow and genetic drift can contribute to changes in cranial shape [5,70–72], the whole skull (cranium plus lower jaws) also represents a highly adapted feature for prey acquisition [73]. Among Canidae, cranial shape varies based on prey size, with long and narrow upper and lower jaws seen in species that hunt small prey, while short and broad jaws are selected for in species that hunt more robust prey [73]. For example, cranial differences were observed between coastal and inland wolves in British Columbia, despite their geographical proximity and the absence of physical barriers to gene flow, owing to differences in habitats and prey [74]. Coastal wolves use marine resources as well as Sitka black-tailed deer (*Odocoileus hemionus sitkensis*), while those on the mainland feed primarily on larger prey such as moose, elk (*Cervus canadensis*), caribou (*Rangifer tarandus*) and Stones sheep (*Ovis dalli stonei*) [75,76]. As such, the cranial characteristics of these two populations probably reflect their diet, with the coastal wolves being smaller than their mainland counterparts [12,74]. In fact, studies on several wolf populations confirm the significant positive relationship between prey size (average prey weight) and skull size (maximum skull length, zygomatic breadth or a series of skull measurements summarized as PC1) [12,14], body size [77] or both [13]. Skull size was also observed to correlate with ambient temperature or precipitation, both of which affect prey composition, which in turn affects carnivore morphology [14,78]. By contrast, cranial shape differences between Dinaric-Balkan and Carpathian wolves seem to have evolved in the absence of differences in main prey; nevertheless, the authors suggest that the effects of prey on skull morphology in these two populations cannot be completely excluded [64].

The main prey of wolves in Fennoscandia is moose [79–82], whose densities reached critically low numbers at the end of the nineteenth and beginning of the twentieth centuries across the entire peninsula [25,26,83]. Since the 1970s onwards, moose numbers have dramatically risen across the region owing to different forestry and management practices [25,26]. Therefore, it is also likely that changes in prey over time could have played a role in the observed differences in cranial morphology of Fennoscandian wolves. Interestingly, low prey density in the past has also been considered one of the driving forces behind documented cases of wolves attacking and consuming humans in Finland at the end of the nineteenth century ([51], but see [16,84]). Both of our sampled Turku wolves had narrow cranial width and small CS, falling within the morphospace of the *historical Finnish* wolf population that experienced low prey densities (highlighted with a red outline in figures 1, 3 and 4). These Turku wolves also preyed upon livestock, which could have facilitated their transition to attacking and consuming children [51].

Another potential contributor to changes in morphology is inbreeding, which can lead to shared morphological features owing to common ancestry among inbred individuals (aside from malformations that can arise under this scenario [85,86]. Although inbreeding and inbreeding depression have been observed, especially in the *modern Scandinavian* population, not all *modern Scandinavian* individuals are equally inbred. Unfortunately, we did not have information on inbreeding for the specimens in our sample. Nevertheless, inbreeding has previously been linked to congenital deformities in the Scandinavian wolf population, including missing teeth (hypodonty), malformed teeth, canines pointing more forward (mesioversion of canines), smaller teeth than normal (microdontia) and more teeth than usual (supernumerary teeth), among other cranial and skeletal anomalies [32]. As our landmarking scheme was designed to capture the overall shape of the cranium and recorded, on average, only one landmark associated with each tooth, we were not able to capture these deformities despite their clear visibility in some samples. However, with a more detailed morphometric approach focussed on teeth and inbreeding data on each individual, it could be possible to test the contribution of this factor to localized morphological changes within Fennoscandian wolves.

Although wolf-dog hybridization can also contribute to changes in morphology, recent hybridization is likely to bring more abrupt changes to the cranial shapes of wolves than prey-driven adaptations or plasticity. For example, the offspring of first-generation wolf-dog hybrids often display intermediate characteristics [87–89]. Because dog breeds display a greater variety of shapes than any other canids, recent hybrids should be detected as outliers in our dataset. We did not observe such cases. While hybridization and introgression of dog DNA into wolves have been observed across the world [90–93], genetic evidence suggests that the level of genetic introgression of dog genomes into Scandinavian wolves is comparable to or even lower than in the other parts of the world [36,94].

The different genetic sources of *historical* and *modern* Fennoscandian wolf populations have probably played important roles in shaping their morphology. At the same time, numerous studies have demonstrated how wolf morphology changes in response to biotic and abiotic factors such as prey, precipitation and temperature. Wolves tend to migrate within similar habitats [95–98], which may help preserve the genetic structure of populations. Given the complexity and potential interactions of these factors, determining the underlying causes of morphological changes in Fennoscandian wolves is challenging. Nevertheless, the influence of prey species on morphology could be further explored using stable isotope analyses. Additionally, incorporating genomic data of the individuals in this study could enable direct testing of the link between genetic and morphological differences. In this case, we could examine the genetic basis for morphological traits, investigate the role of genetic drift in shaping morphology, quantify genetic introgression and how it is reflected in morphology, and relate inbreeding effects to morphological changes.

## 5. Conclusion

In recent centuries, humans have significantly influenced the population dynamics of numerous species through actions such as hunting, the introduction of non-native species and habitat alteration. Our study provides evidence of morphological changes coinciding with the genetic replacement of Fennoscandian wolf populations, which were driven to near extirpation during the twentieth century owing to severe persecution. Our results also suggest that the changes in prey composition and the founder effect may have influenced cranial traits in the *modern Scandinavian* population, which displayed different mean shapes compared to the *modern Finnish* population. Future research on wolf populations could integrate genetic, ecological and morphological data, particularly by using three-dimensional landmarking techniques that capture subtle phenotypic changes and by including tooth shape. This comprehensive approach holds promise for uncovering the genetic underpinnings of morphological variation and the factors driving such transformations, including those caused by humans.

## Data Availability

The landmark data and R code are available from the Dryad Digital Repository [99]. Supplementary material is available online [100].
